# Diagnostic Features and Subtyping of Thymoma Lymph Node Metastases

**DOI:** 10.1155/2014/546149

**Published:** 2014-07-08

**Authors:** Stefano Sioletic, Libero Lauriola, Enzo Gallo, Robert Martucci, Amelia Evoli, Giovannella Palmieri, Enrico Melis, Giuseppe Pizzi, Massimo Rinaldi, Maurizio Lalle, Edoardo Pescarmona, Pierluigi Granone, Francesco Facciolo, Mirella Marino

**Affiliations:** ^1^Department of Pathology, Regina Elena National Cancer Institute, Via Elio Chianesi 53, 00144 Rome, Italy; ^2^The Pennine Acute Hospitals NHS Trust, Royal Oldham Hospital, Rochdale Road, Oldham, Manchester OL1 2JH, UK; ^3^Catholic University of Rome, Largo Francesco Vito 1, 00198 Rome, Italy; ^4^Department of Neurosciences, Catholic University of Rome, Largo Francesco Vito 1, 00198 Rome, Italy; ^5^Department of Molecular and Clinical Endocrinology and Oncology, “Federico II” University, Via Pansini 5, 80131 Naples, Italy; ^6^Thoracic Surgery, Regina Elena National Cancer Institute, Via Elio Chianesi 53, 00144 Rome, Italy; ^7^Radiology , Regina Elena National Cancer Institute, Via Elio Chianesi 53, 00144 Rome, Italy; ^8^Medical Oncology “B”, Regina Elena National Cancer Institute, Via Elio Chianesi 53, 00144 Rome, Italy; ^9^Medical Oncology, S. Eugenio Hospital, P. le dell'Umanesimo 10, 00144 Rome, Italy; ^10^Division of General Thoracic Surgery, Catholic University of Rome, Largo Francesco Vito 1, 00198 Rome, Italy

## Abstract

*Aim.* The purpose of the present study was to characterize the morphological features of thymoma metastases in lymph nodes and to evaluate the possibility of their subtyping according to the 2004 WHO classification of thymus tumors.* Materials and Methods.* We reviewed 210 thymoma cases in our series of thymic epithelial tumors (TET), including their recurrences and lymphogenous metastases. Three cases of lymph node metastases, one case occurring synchronously with the primary tumor and one synchronously with the first relapse (both in intrathoracic location) and one case of metastasis observed in a laterocervical lymph node subsequently to two thymoma relapses were found.* Results.* The metastatic nodes were variably but extensively involved in all cases. The histological features were similar in both primary tumors and metastases. Thymoma metastases were subtyped according to the WHO classification as B3 (one case) and B2 (two cases), and distinctive features in comparison to metastatic epithelial neoplasias from other sites were observed.* Conclusion.* Thymoma lymph node metastases, although rare, can be subtyped according to the WHO classification on the basis of their morphological and immunohistochemical features. Clinically, the presence of nodal metastases may herald subsequent relapses and further metastases even in extrathoracic sites.

## 1. Introduction

Thymic epithelial tumors (TET) with organotypic (thymus-like) features (thymomas) may be considered relatively benign in limited stages (I-II according to Masaoka et al.) [1]. In advanced stages mostly B2 and B3 thymomas and thymic carcinomas (TC) show local relapse and/or pleural dissemination, lung metastases, and/or great vessel involvement [2, 3]. Lymphatic and hematogenous metastases in thymoma are a rare event which is conversely more frequently observed in thymic carcinomas [4]. Only two out of the 207 thymoma cases reported in the series by Yamakawa et al. [5] developed extrathoracic lymphatic dissemination (neck lymph nodes). In 2012, Vladislav et al. [6] reported, on a series of 13 cases of thymoma metastatic in extrathoracic location, only one case of B3 thymoma showing lymph node metastasis in axillary lymph nodes, concurrently with multiple localizations in extrathoracic sites. In fact, a possible origin from silent and/or known previous thymoma should always be considered for unusual lymphoepithelial or epithelial metastases occurring in the thorax/mediastinal lymph nodes or even in extrathoracic sites. The morphologic features of lymph node metastases in thymoma have rarely been described [7]. In our series of 210 thymoma cases, lymph node metastases were found in three cases. The lymph node metastases had an intrathoracic location in two cases at N2 Level according to Yamakawa et al. [5] and an extrathoracic location (laterocervical, N3 Level) in one case. The intrathoracic lymph node metastasis was synchronous with the primary tumor in one case, whereas in the other two cases the lymph node metastasis occurred 5 years (intrathoracic) and 9 years (neck lymph node) after the original diagnosis, respectively. In all cases the histological features of lymph node metastases were similar to the primary tumor, and distinctive morphological features were found in comparison with epithelial metastases from other sites.

## 2. Materials and Methods

### 2.1. Case Reports

Three thymoma cases with intrathoracic (2 cases) or extrathoracic (1 case) lymph node metastases were found in a series of 210 consecutive thymoma cases at the Regina Elena National Cancer Institute and the Catholic University of Rome, occurring in the years 1994–2012. TC (most of them squamous cell carcinoma) and thymic neuroendocrine carcinoma (TNEC) were mostly diagnosed on bioptic material, were in advanced stage (III or IV), and were treated with neoadjuvant or adjuvant chemotherapy; no metastatic lymph node specimens were available among surgically removed TC, TNEC, or their metastases.

**Case 1 .**

In 2006, a 42-year-old female, with no myasthenia gravis (MG), had a recurrence of thymoma 5 years after the first diagnosis of B3 thymoma (WHO), stage III (Masaoka). In the relapsed tumor a B3 thymoma was confirmed, with focal features of well differentiated thymic squamous carcinoma (combined B3/squamous carcinoma). Moreover, a “pleural” nodule was found to be histologically consistent with a subpleural lymph node metastasis of B3 thymoma. In 2008, the patient had another costodiaphragmatic nodular recurrence of B3 thymoma. She later developed a renal cell carcinoma. The patient was lost to follow-up.

**Case 2 .**

In 1994, a 37-year-old female underwent a surgical resection of stage III thymoma with pleural implants 1 year after a diagnosis of B2/B3 thymoma, treated with neoadjuvant chemotherapy. No MG was associated. During the operation the surgeon found an enlarged intrathoracic lymph node (station 10). Both the tumor tissue and lymph node metastasis were diagnosed as B2 thymoma. Subsequently, the patient developed multiple paravertebral relapses and distant metastases in the liver, bone, and lungs. Several conventional and nonconventional therapeutical regimens were applied. The patient died in 2009 due to infectious complications, free of thymoma relapse, 15 years after the original diagnosis. Therapeutical aspects of this case have already been reported [8].

**Case 3 .**

In 1999, a 49-year-old female had a mediastinal mass excised and diagnosed as a B2 thymoma, stage III (according to Masaoka). No MG was associated. The patient had a recurrence of the primary tumor, with costal involvement in 2006 and a second lung and pleural relapse in March 2008. An enlarged laterocervical lymph node was excised in July 2008 with a histological diagnosis of B2 thymoma metastasis. In spite of the advanced disease phase, the patient did well for several years after the biopsy without other relapses or metastases. However, she died recently (2013) because of surgical complications due to pericardial thymoma relapse, after 14 years of follow-up (FU).

### 2.2. Materials and Methods

The institutional review board approval was obtained for our study. In all cases the primary tumor and the metastases were available. In Cases 1 and 3 formalin-fixed, paraffin embedded tissues were available for the morphological and immunohistochemical study. In Case 2, a few tissue slides were available. All tumours were classified according to the 2004 WHO classification of thymus tumors [9]. The immunophenotype of both epithelial cells (EC) and lymphoid cells was assessed on formalin-fixed, paraffin embedded tissue by immunohistochemistry (IHC). The following antibodies were utilised: CD3, CK19 (clone RCK108), and CD1a (clone 010) from Dako (Milan, Italy); CD5, TdT (clone SEN28), and CD117 (clone T595) from Novocastra (Menarini, Florence, Italy). Unmasking of antigenic sites was performed by treatment of the sections in a thermostatic bath at 96°C for 40 min in citrate buffer (pH 6); for CD5 slides were pretreated in EDTA buffer (pH8). Immunostaining was revealed by a streptavidin-biotin enhanced immunoperoxidase technique (Super Sensitive MultiLink, Novocastra, Menarini, Florence, Italy) in an automated stainer (Bond Max, Menarini). Diaminobenzidine (Menarini) was used as a chromogenic substrate.

## 3. Results

**Figure 1:**
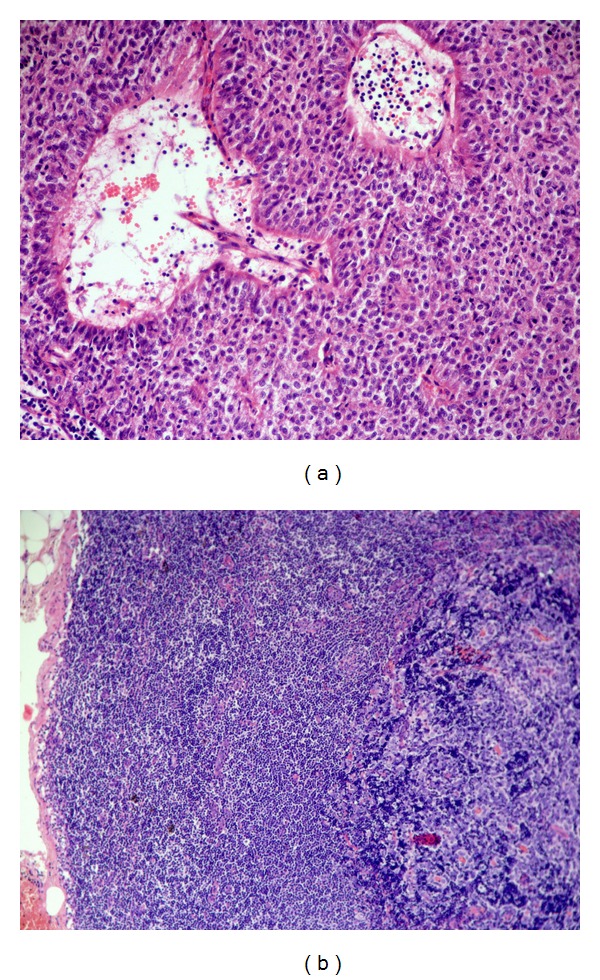
(a) Case 1, HE, 200x. B3 thymoma metastasis in the lymph node. EC with scant cytological atypia are seen forming sheets and palisades around vessels. Two perivascular spaces (PVS) are seen. (b) Case 3, HE, 50x lymph node partial involvement by metastatic B2 thymoma. The subcapsular sinus is partially preserved. The immature T-lymphocyte-rich metastasis of B2 thymoma is seen on the right, whereas residual lymph node B cell follicles and T-cell areas are seen on the left.

**Figure 2:**
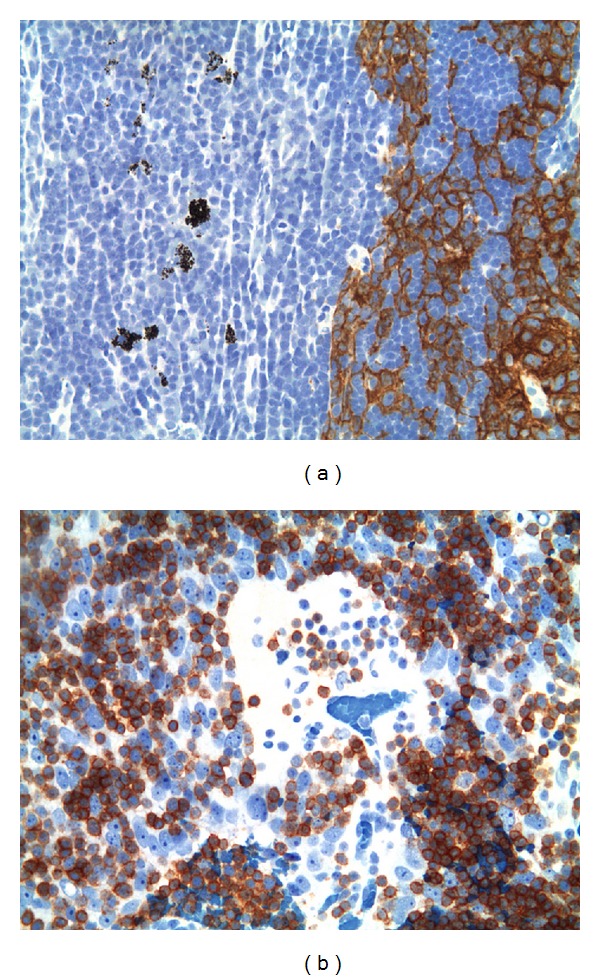
(a) Case 3, 200x. CK19 staining of EC networks in the laterocervical lymph node, sharp distinction to the normal lymph node. Scattered macrophages with granular dust are seen on the left in the normal part of the lymph node. (b) Case 2, 400x. CD1a + immature T cells around and in the PVS formed in the lymph node metastasis among EC.

The lymph node involvement in Case 1 was subtotal; in Cases 2 and 3 the lymph node involvement was partial. Particularly, in Case 3 the preoperative ultrasonographic study of the laterocervical node suggested a partial lymph node involvement due to slight lymph node hilus displacement. US power Doppler evaluation showed poor and confused vascularisation within the nodule; the lymph node hilum was not clearly seen. In Case 1, the metastasis showed the features of type B3 thymoma, with EC growing in sheets and nests forming palisades around vessels, similar to the perivascular spaces (PVS) (Figure 1(a)). Few lymphocytes of both mature (CD5+/CD1a−/Tdt−) and immature (CD1a+, Tdt+) T cell phenotypes were seen scattered among the EC or in the perivascular spaces (PVS). The tumor expressed the typical keratin 19 positivity of thymoma; no CD5 or CD117 positive EC were found. In Cases 2 and 3, the lymph node architecture was partially preserved and the metastases were very similar to the primary B2 thymoma. No significant morphological differences were found in comparison with the primary tumor with respect to the neoadjuvant chemotherapy given in Case 2. In both cases, the metastasis and the normal lymph node were seen closely intermingled, without a plasmacellular reaction neither a fibrotic response surrounding epithelial nests (Figure 1(b)). In all cases, the formation of PVS, a typical feature of B2 thymoma and B3, was frequently observed. The scattered EC of the tumor and the scattered histiocytes conferred to the involved part of the lymph node, a “starry sky” pattern (Figure 1(b)). In Cases 2 and 3, IHC demonstrated both the EC nature, CK19 positive of the scattered “clear” cells (Figure 2(a)), and the immature CD1a+ phenotype of the T-cell rich areas, as well as the occurrence of immature T-cells in PVS (Figure 2(b)).

## 4. Discussion

Lymphogenous metastases of thymoma are very rare. Yamakawa et al. [5], in a study on 207 cases of thymoma with an adequate follow-up, found a total of 5 lymphogenous metastases, but no correlation was made with the histology of the thymoma. Kondo and Monden [4], in a study on 1320 patients with thymic neoplasms, found that the rate of lymphogenous metastases in thymoma, thymic carcinoma, and thymic carcinoid was 1,8%, 27%, and 28%, respectively. The survival rate of thymoma patients with metastases to intrathoracic lymph nodes was worse than those with metastases limited to anterior mediastinal lymph node, although no significant statistical difference was found. On the contrary, a significant difference in survival rate was observed in thymic carcinomas with or without lymph node metastases. Although our series was limited in number, we focused on thymoma metastases to lymph nodes as it appears that scant literature data are available describing in detail the morphological features of metastases. This is of concern as it is well known that thymoma patients are at risk for a second malignancy [10]. Moreover, the recurrence and/or metastases of thymoma could develop also after a long disease-free survival (DFS), increasing the differential diagnostic problems. In our thymoma series, the lymph node metastases occurred at or following a relapse, and in two cases the lymph node metastasis heralded further relapses (Case 1) or distant (Cases 1 and 2) metastatic spread. The differential diagnosis of metastatic B1-B2 thymomas includes small round blue cell tumors and, in case of a B3 thymoma, the low-grade renal cell carcinoma [6]. In our series of three cases, lymph node metastases were of B-type consistent with the more aggressive behaviour of B-type thymoma [3] and the morphological hallmarks of B-type thymoma; that is, PVS and perivascular palisades of EC [9, 11] were retained in the lymph node metastases. Our observations confirm previous scarce reports on the similarities between primary and metastatic lesions of thymoma. No decrease in the lymphocyte population was found at variance with other extrathoracic metastases described [6]. So far, thymoma lymph node metastases have rarely been subtyped, after the one case described by Salter and Krajewski in 1986 [7], who described a lymphocytic thymoma, probably corresponding to a B2 thymoma metastasis. A further report from Barat et al. [12] described 3 cases of metastatic involvement of neck lymph nodes by “malignant thymomas” of epithelial type. However, their description of multiple mitotic figures and of necrosis occurring in EC in lymph nodes might better correspond to TC cases. By immunophenotyping of our cases, neither CD5 surface molecule, typically expressed by TC, mainly of squamous cell type [13–15], nor CD117, a useful TC marker [16, 17], was found to be expressed. A differential diagnosis of thymoma metastasis* versus* lymph node involvement by T-lymphoblastic lymphoma was considered for Cases 2 and 3: MIC-2 antibody (CD99) and TdT proved useful in assessing an immature phenotype of lymphocytes present in lymph node metastases, while the anti-CK 19 staining was useful in confirming the epithelial nature of the metastatic lymph node infiltration. Moreover, in our cases, the immature T-cell population comprised a wide spectrum of variably sized cells and neither atypia nor necrosis was seen, thus excluding the possibility of a T lymphoblastic lymphoma. In this respect, Inoue et al. [18] by flow-cytometry found that the phenotypic characteristics of lymphocytes in thymoma metastases were similar to those in the primitive tumor. However, in their study, T cells appeared to be less mature than those in the primary lesions, suggesting that the functional characteristics of EC may be deficient and different in metastases* versus* primary tumors. We showed here that IHC plays a major role in defining the epithelial nature of unclear lymph node metastases with a high immature T cell content. Moreover, we also demonstrated that in our cases a careful morphological examination of the metastatic lesions could reveal their thymic origin, as the organotypic (thymus-like) features were preserved in a specific and characteristic pattern. However, our observations on a limited case series should be validated by further reports. It is noteworthy that our thymoma cases with lymph node metastases showed a long natural history. In Case 1, a renal cell carcinoma, occurring 8 years after the primary diagnosis, could be responsible for the subsequent oncological history of the patient; we lost her to our FU. In Case 2, the final event occurred after 15 years and several metastases; the death was not linked to further tumor recurrence and/or metastasis but to infectious complications. In Case 3 the metastasis, occurring in a N3 location according to the Yamakawa et al. [5] system of lymph node subgrouping for thymic malignancies, was not followed by clinical worsening, and the patient did well until very recently; then a fatal pericardial relapse occurred (third relapse), 14 years after the original diagnosis. The long clinical histories of our cases highlight the fact that, besides anatomical staging, biological prognostic features (still largely unknown) could be relevant in thymoma patients. Recent data point to the relevance of lymph node dissection and reporting in thymic epithelial malignancy [19]. Lymph node metastasis of thymoma, although rare, is now subjected to renewed interest in the framework of the upcoming specific official TNM staging system for TET for the eighth edition of the American Joint Committee on Cancer/Union for International Cancer Control (AJCC/UICC) [20, 21]. Future prospective studies including careful lymph node assessment in thymoma will provide further insight into the natural history of these rare tumors.
